# Acquired Melanonychia in Chilean Patients with Essential Thrombocythemia Treated with Hydroxyurea: A Report of 7 Clinical Cases and Review of the Literature

**DOI:** 10.1155/2013/325246

**Published:** 2013-02-07

**Authors:** Nigel P. Murray, Pablo Tapia, Jose Porcell, Maximiliano Echavarria, Hernán Suazo

**Affiliations:** ^1^Hematology Department, Hospital de Carabineros, Simon Bolıvar 2200, Nunoa, 7770199 Santiago, Chile; ^2^Faculty of Medicine, University Mayor, Renato Sanchez 4369, Las Condes, 27550224 Santiago, Chile; ^3^Division of Medicine, Hospital de Carabineros, Simon Bolıvar 2200, Nunoa, 7770199 Santiago, Chile; ^4^Faculty of Dentistry, University de Desarrollo, Avenida Las Condes 12.438, Lo Barnechea, 27470325 Santiago, Chile; ^5^Pharmacy Department, Hospital de Carabineros, Simon Bolıvar 2200, Nunoa, 7770199 Santiago, Chile

## Abstract

Longitudinal melanonychia has been associated with a range of drugs, especially chemotherapeutic agents. We report 7 cases of melanonychia associated with the use of hydroxycarbamide for essential thrombocythemia. Of a patient population of 27, 7 (26%) developed melanonychia over a period of 2–7 years, and was not dose dependent. The high incidence of melanonychia in Chilean patients may be in part due to their Hispanic descent or to the high levels of UV radiation found in Santiago.

## 1. Introduction

We present 7 cases, five men and two women, all with a diagnosis of essential thrombocythemia, who presented with longitudinal dark pigmentation of the nails after a period of 3 to 5 years of treatment with hydroxyurea. Acquired longitudinal melanonychia is characterized by the presence of longitudinal brown or black lines in the nail plate as a result of increased melanin deposits. They originate in the nail matrix and are the result of an increased production of melanin by matrix melanocytes or of an increased number of melanocytes in the nail matrix.

## 2. Patients

Of 27 patients currently being followedup for essential thrombocythemia and being treated with hydroxycarbamide, 7 (26%) patients have developed melanonychia. 

The clinical details of the 7 patients are shown in Table 1, with a mean age of 71.7 ± 9.8 years, and all the patients were treated with hydroxyurea for essential thrombocythemia for a median time of 5 years (range 2–7 years) and a median dose of 1,500 gm/day (range 500–2500 mg/day).

All patients did not have significant comorbidities and the use of other drugs was limited to aspirin 100 mg and in four patients the use of allopurinol 100 mg/day.

**Figure 1:**
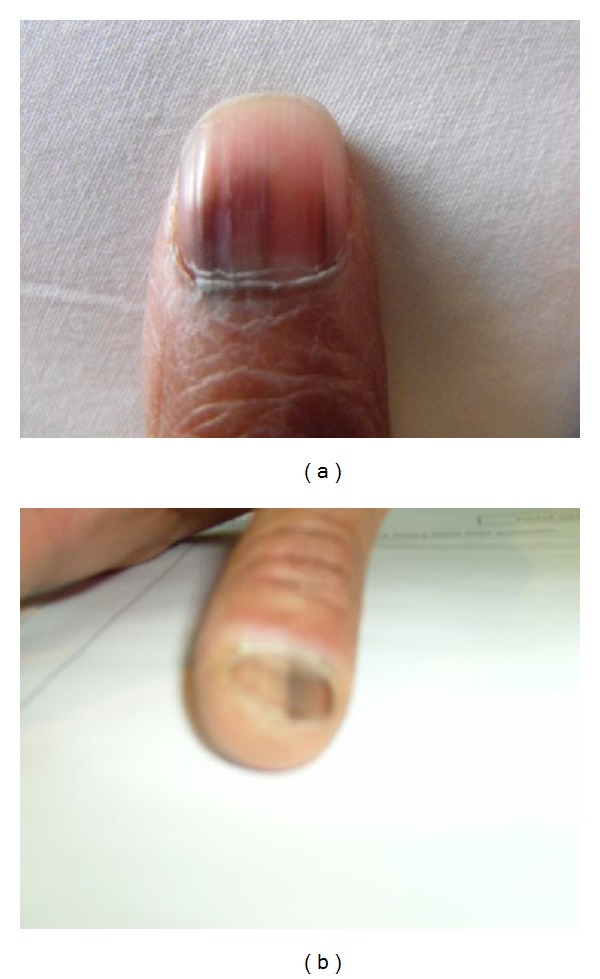
(a) Close of longitudinal melanonychia. (b) Melanonychia of the thumb nail.

Nail examination (Figures 1(a) and 1(b)) showed a dark brown pigmentation distributed in well-defined longitudinal lines of varying width, and the nail was smooth and shiny. The lesions themselves were asymptomatic and Hutchinson's sign was negative. With time, the lines occupied the full length of the nail. In two patients, there was a generalized hyperpigmentation of the skin. The number of nails affected was variable as was between nails of the hands and feet. There was no relation between the duration of hydroxycarbamide therapy or the dose used to control the essential thrombocythemia. 

## 3. Controls

- Seven patients (6 men and 1 woman) with polycythemia rubra vera and treated with venesection for a median of 5 years were evaluated for melanonychia, and none of the 7 patients showed this condition.
- Fifty patients with atrial fibrilation attending an outpatient oral anticoagulant Clinic were also evaluated to assess the frequency of melanonychia in a general Chilean population, and 3 (6%) of these patients were identified as having this nail condition. The melanonychia involved 3 to 4 nails and was found in the hands but not in the feet.

## 4. Discussion

A healthy adult has approximately 200 melanocytes per mm^2^ in the nail matrix, of which the majority remain dormant [1]. When these melanocytes are activated, melanosomes filled with melanin are transferred to differentiating matrix cells, which migrate distally as they become nail plate onychocytes [1]. This results in a visible band of pigmentation in the nail plate. The prevalence of affected individuals increases with age [2, 3], and there is a physiological or a racial component, being more common in African Americans or in those of Hispanic origin [4, 5]. The prevalence of melanonychia in the general population has been estimated to be 1%, increasing to 12% in hospitalized patients [6].

The largest reported series of melanonychia in patients with essential thrombocythemia and associated with hydroxycarbamide is of 9 cases [7], there was no association with the dose or duration of treatment, and equally could affect the nails of both the hands and feet and could be associated or not with other dermatological changes such as skin hyperpigmentation. The same characteristics were observed in our series of 7 patients. Differing from our general outpatient population, in patients treated with hydroxycarbamide, the melanonychia occurred in both hands and feet, although more nails seemed to be affected in the hands. 

Hydroxycarbamide is a cytostatic agent used in the treatment of myeloproliferation, inhibiting cellular DNA synthesis and promoting cell death in the S phase of the cell cycle [6]. It appears that hydroxycarbamide causes melanonychia by melanocytic activation. This is a process where there is increased melanic pigmentation of the nail matrix epithelium and nail plate without an increase in the number of melanocytes [8]. Although decreasing the dose or discontinuing the use of hydroxycarbamide could eliminate with time the melanonychia [8], the chronic nature of the underlying myeloproliferation and the few alternative treatments make a therapeutic change difficult. This adverse event is not considered sufficient to stop hydroxycarbamide treatment in patients with essential thrombocythemia, according to a unified definition of clinical resistance or intolerance to hydroxycarbamide; however, it may precede the appearance of more serious mucocutaneous side effects such as skin ulceration or the development of skin carcinoma [9]. 

It has been postulated that one cause is photosensitivity, and in Santiago, Chile, the levels of UV radiation are high during most of the year, and this may be a contributing factor for the relatively high incidence of melanocytic in the patients taking hydroxycarbamide, apart from the racial influence of being of a Hispanic origin. The high UV radiation levels may also explain why the melanonychia was more common in the hands than in the feet and was only found in the hands of the control patients, reflecting total UV radiation exposure. 

In the reported cases, it has been estimated that the risk of developing melanonychia in patients receiving treatment with hydroxycarbamide is 4%, more commonly in women and after a long period of treatment [10].

Longitudinal melanonychia of a single nail unit in an adult is concerning, and a biopsy of the nail unit can evaluate the possibility of melanoma, which cannot be differentiated from benign causes of longitudinal melanonychia solely based on clinical examination [11]. When nearly all the nails are affected in patients taking hydroxycarbamide, the diagnosis is consistent with melanonychia; however, it may be necessary for a biopsy in order to exclude acral lentiginous melanoma.

## Figures and Tables

**Table 1:** Clinical features of nail pigmentation.

Age	Sex	Mean dose/day	Time using hydroxyurea	Number of nails affected hands (feet)	Skin hyperpigmentation
78	M	1500 mg	2 years	3 (0)	No
84	M	2500 mg	5 years	10 (10)	Yes
63	M	500 mg	6 years	2 (0)	No
59	M	1000 mg	3 years	5 (2)	No
81	M	500 mg	4 years	10 (10)	No
64	F	1500 mg	5 years	8 (4)	No
73	F	2000 mg	7 years	6 (10)	Yes
